# Effect of Light Emitting Diodes (LED) Exposure on Vitreous Metabolites-Rodent Study

**DOI:** 10.3390/metabo13010081

**Published:** 2023-01-03

**Authors:** Nagarajan Theruveethi, Manjunath B. Joshi, Judith S. Jathanna, Manna Valiathan, Shama Prasada Kabekkodu, Manasa Bhandarkar, R. Huban Thomas, Rajesh Thangarajan, Shailaja S. Bhat, Sudarshan Surendran

**Affiliations:** 1 Department of Optometry, Manipal College of Health Professions, Manipal Academy of Higher Education, Manipal 576104, India; 2 Department of Ageing Research, Manipal School of Life Sciences, Manipal Academy of Higher Education, Manipal 576104, India; 3 Kasturba Medical College Manipal, Manipal Academy of Higher Education, Manipal 576104, India; 4 Department of Cell and Molecular Biology, Manipal School of Life Sciences, Manipal Academy of Higher Education, Manipal 576104, India; 5 Department of Anatomy, International Medical School, Management and Science University (MSU), Shah Alam 40100, Malaysia; 6 American University of Antigua College of Medicine, University Park, Jabberwock Beach Road, Coolidge P.O. Box 1451, Antigua and Barbuda

**Keywords:** vitreous, LED, amino acids, metabolites, visual system, chronic light

## Abstract

The exposure to blue and white Light emitting diodes (LED) light leads to damage in the visual system with short-term LED light exposure. Chronic exposure, adaptive responses to light, and self-protective mechanisms against LED light exposures need to be explored, and it would be essential to understand the repercussions of LED radiation on vitreous metabolites. A total of 24 male Wistar rats were used in this study, divided into four groups (*n* = 6 in each group). Three experimental groups of rats were exposed to either blue, white, or yellow LED light for 90 days (12:12 light-dark cycle routine) with uniform illumination (450–500 lux). Standard lab settings were used to maintain control rats. Vitreous fluids were subjected to untargeted metabolomics analysis using liquid chromatography-mass spectrometry (LC/MS). PLS-DA analysis indicated significant the separation of m metabolites among groups, suggesting that LED exposure induces metabolic reprogramming in the vitreous. Amino acids and their modifications showed significant alterations among groups which included D-alanine, D-serine (*p* < 0.05), lysine (*p* < 0.001), aspartate (*p* = 0.0068), glutathione (*p* = 0.0263), taurine (*p* = 0.007), and hypotaurine. In chronic light exposure, the self-protective or reworking system could be depleted, which may decrease the ability to compensate for the defending mechanism. This might fail to maintain the metabolomic structural integrity of the vitreous metabolites.

## 1. Introduction

A non-ionizing visible light spectrum between 380 and 700 nm plays a significant role in visual perception [1]. Environmental light plays a significant role in zeitgebers [2,3]. The retinal ganglion cell layer and photoreceptors provide vital information about ambient illumination and light-dark cycles to visual and non-visual higher cortical centers [4]. Non-natural light is an essential source in contemporary life; a large segment of the population is exposed to different types of light-emitting diodes (LEDs), and it is used globally for interior and exterior lighting and rudimentary lighting components of digital devices.

The blue range of the visible spectrum has high energy that can penetrate tissues, which is associated with the occurrence of malignant melanoma compared to other wavelengths of the visible spectrum in animal models [5]. LED radiation diminishes the capability of cellular mechanisms and increases apoptosis, damaging DNA through excessive ROS production [6,7]. These mechanisms are activated by chromophores within the retinal cells and are intrinsically sensitive to retinal ganglion cells [8]. These LEDs can alter the function of ocular (retinal) and other biological tissues [4,6,9,10]. Excessive exposure to light can alter physiological rhythms, such as sleepiness and alertness and increase cortisol levels [11,12].

Radiation-induced retinal damage can occur either through photo-vaporization or photochemical mechanisms [13,14]. Photo-vaporization results in tissue disruption by short-wavelength light exposure with a high irradiance level [15]. The photochemical injury occurs when tissue temperature increases due to the entrapment of thermal energy into a tissue molecule, which could result in retinal damage [16]. Biological chromophores of retinal pigmented epithelial cells can absorb the photons of light from the visible spectrum 380–780 nm. These absorptions are necessary for visual functions, but peak energetic radiations (from violet to blue, 400–500 nm) can lead to a lethal consequence for retinal cells [17,18]. By dissipating the excess energy, these absorbed excited states of electrons can return to the inhibited state. The energy molecules can break a bond in another molecule through a direct exchange of electrons or hydrogen-producing reactive oxygen species (ROS) [10,19]. The interaction of these chromophores with photons of light might generate ROS, damaging amino acids, lipids, and DNA [20].

The visual system is exposed for a limited time to LED lights of different spectra as its technology is blooming to decrease energy consumption and CO_2_ emissions. These technologies eventually replaced standard lighting systems indoors and outdoors. The white and blue LEDs have high energetic characteristics compared with other traditional light sources, so the potential ocular and general risks of these LEDs are widely explored [4,19,21,22,23,24,25]. The short-term (28 days) exposure to moderate blue light causes nebulous metabolites in the vitreous [26] and changes in retinal tissues and cortical neurons [27,28]. Inference from these studies cannot be generalized because few studies have suggested that low levels of blue light for a longer duration (a few months to years) are needed to determine the effects of blue light on the visual system [29]. Considering this, we have hypothesized that chronic exposure to light might develop a self-protective mechanism, stimulated by an increased insult to ocular fluid. Hence, we aimed to understand the relationship between chronic light exposure and alterations in the vitreous metabolites in Wistar rats.

## 2. Methodology

**Figure 1:**
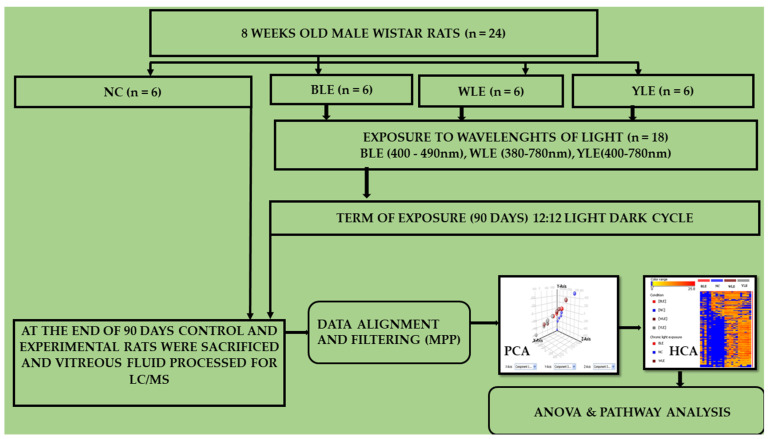
Explains the methodological flow and data analysis ofnormal controls (NC), blue light exposure (BLE), white light exposure (WLE), and yellow light exposure (YLE) groups.

The study used 8-week-old healthy male Wistar rats (*n* = 24). The rats were divided into three experimental groups that included blue light exposure (BLE), white light exposure (WLE), and yellow light exposure (YLE) groups (*n* = 6 in each group), one serving as a (unexposed) normal control group (NC) (*n* = 6). We have included healthy rats for the study, and the control rats were selected from the same pool. However, it’s safe to assume that the experimental control rats will not have any pathology (Figure 1 explains the methodological flow). Both eyes of the rats were exposed to blue, white, and yellow LED lights with the maintenance of homogeneity and intensity of the lights. The light standard was measured and standardized using Asensetek Lighting Passport Pro, New Taipei City, Taiwan [30]. The spectral sensitivity of different wavelengths of the visible light spectrum (380–780 nm) was used for this experiment (450–500 lux). The spectral properties of light transmittance of the front surface of light, scotopic sensitivity, flicker index, and melatonin suppression of each light were measured. The height from the source to the animals (cage) was 50 cm, and the exposure time (12:12 dark and light cycle routine for 90 days) matched the nocturnal time of the rodents. In the light exposure group, animals were subjected to blue LED (400–490 nm), white LED (380–780 nm), and yellow LED (400–780 nm) light. The relative peak intensity of (420–470) spectral curves for the blue light was seen in the white light and declined/flattened the peak at a similar relative intensity at the yellow light (Supplementary Figure S1). The light source was connected to the top of the cage (L = 100 cm, W = 70 cm, and H = 50 cm). The mounting of the light kept it at 50 cm (100% system level light output at standard operating voltage range, the total light output of the cage at the testing area (450–500 lux). Following exposure, all animals were euthanized by intraperitoneal injection of pentobarbital (i.p. 100 mg/kg; Euthasol^®^) and xylazine (10 mg/kg; Proxylaz^®^) at a lethal dose. Then, eyes were enucleated by using watchmaker forceps (number 5) and Sklar’s blunt enucleation scissors. Immediately after enucleation, the vitreous was extracted by the aspiration (21-gauge hollow needle) technique and stored at −20 °C for LC-MS analysis.

### 2.1. Rat Whole Vitreous

For the chronic light exposure model analysis, the whole vitreous sample was collected by the aspiration technique. The samples were sonicated and stored at −20 °C until LC-MS analysis.

### 2.2. Vitreous Metabolomic Analysis

Untargeted metabolomics was used to analyze the vitreous fluid samples using an LC-MS Agilent LC-QTOF system (Agilent Technologies, Santa Clara, CA, USA). It consisted of an Agilent 1200 LC system coupled online with an Agilent 6520 time-of-flight mass spectrometer. The vitreous samples were defrosted on ice, vortexed, and centrifuged at 12,000 rpm for 15 min. An 8 µL aliquot was injected into an Agilent 1290 LC system coupled with an ESI-Q-TOF instrument (Agilent 6520, Agilent Technologies, Santa Clara, CA, USA). The HPLC column (Phenomenex, Torrance, CA, USA (P/No:00G-4601-E0; Desc: Kinetex 5 μm C18 100A; Size: LC Column 250 × 4.6 mm; S/No H18-343854; B/No 5701-0060) was maintained was 25 °C. The injected vitreous data were collected in the positive mode of the electrospray ionization (ESI) technique. Basic and neutral metabolites were eluted in the positive mode using a subsequent gradient at 400 µL/min using mobile phase A: 0.1% formic acid in water and mobile phase B: 0.1% formic acid in 90% acetonitrile (2% to 98% B in 25 min, 98% B for 10 minutes, and equilibrated to 2% B for 10 min). The ESI spray voltage was maintained at 3.5 kV with the MS interface capillary at 350 °C. The Fragmentor was set at 140 V. Drying gas and a nebulizer were maintained at 8 liters/minute and a pressure of 40 psig, respectively. Data was acquired at an acquisition rate of 2 Hz in a range of 50–1700 m/z. An untargeted mass spectrometer approach was employed based on the precise mass value and retention time to confirm the most abundant and significant metabolites.

### 2.3. Data Processing and Analysis

The LC-MS spectral region of 5.00 ppm was segmented into bins of 0.05 ppm with the Agilent pro software. The region with anything above 5.00 ppm error was excluded from the analysis, as it might have a remnant noise signal. A total column (bins) of metabolites was obtained, and the integrated area within each bin was normalized to a constant sum of 500 for each range to minimize the effects of variable concentration among different samples.

**Figure 2:**
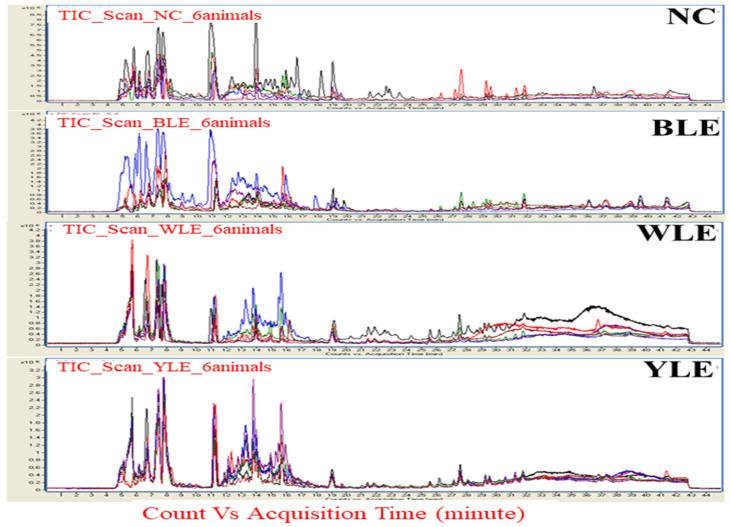
Representation of the spectral features of metabolite peaks obtained in controls (NC), blue light exposure (BLE), white light exposure (WLE), and yellow light exposure (YLE) groups separately. The spectral color of the chromate gram represents each animal from the same group. The *X*-axis represents the count versus acquisition of the metabolites’ time in minutes, and the *Y*-axis represents the generated distinct peaks on the total ion chromatogram (TIC) traces (×10^6^).

The raw data from each run was processed using a molecular feature extraction tool in the Qualitative Mass Hunter Analysis Software B.04.00 (Agilent Technologies, Santa Clara, CA, USA). The data files containing monoisotopic mass, respective abundance, and retention time were used for data alignment and filtering in the Mass Profiler Plus software (MPP) (Agilent Technologies, version B.12.5). Raw data files were segregated, aligned, transformed to log10, and baselined to the median of all samples in MPP. The features present in at least 75% of individuals in each group were considered for further analysis. A partial least square discriminant analysis (PLS-DA) was performed on the metabolome data to discriminate between the study groups. Compounds were identified in the METLIN and HMDB databases based on isotopic pattern distribution and accurate mass within a specified tolerance (15 ppm error). Peaks obtained from the raw MS data were aligned and subjected to bioinformatics and statistical analysis. The metabolite peaks were obtained from normal controls (NC), blue light exposure (BLE), white light exposure (WLE), and yellow light exposure (YLE) groups separately (Figure 2).

## 3. Results

### 3.1. Metabolomic Amendment of Chronic Light Exposure and Reprogramming

The vitreous fluid aspiration was conducted at 10:00 AM Indian Standard Time (IST) in order to maintain diurnal time. A total of 24 vitreous samples withdrawn from light exposure (BLE, WLE, and YLE) and the control group were analyzed. We identified 20,884 putative library compound matches across the groups, and these compounds were filtered out by frequency. Out of these, 459 compounds were presented across the samples. Out of the obtained compounds, 243 were statistically significant (*p* < 0.05) for the entity of a 2.0-fold change (FC). Further, we analyzed differential expression using a t-test against zero and kept the *p*-value asymptotic across the samples with an FC of 3.1. Out of a total of 20,884 molecules, 1521 were statistically significant to specific 907 molecules (*p* < 0.05), 348 molecules (*p* < 0.02), 169 molecules (*p* < 0.001), 82 molecules (*p* < 0.0001), and 20 molecules (*p* < 0.00001), where they exhibited definite statistical alteration.

### 3.2. PCA and PLS-DA

We applied the principal component analysis (PCA) to all detected metabolites. The grouping of the NC, BLE, WLE, and YLE groups demonstrated their metabolomic expression differences. Most metabolites with extreme abundance were indeed isolated in light groups. Further, we created a summary of frequency normalization for abundance values (log10) across the groups; out of it, 99% of the samples were normally distributed, and outliers were removed in each group. Only significant metabolites were taken into account; the molecules in the same group clustered with each other, showing less difference among the light exposure group.

We found the pattern of metabolomics through partial least squares discriminant analysis with a sensitivity of 90% and a specificity of 87%. The amino acid and small molecule levels were notably elevated in the BLE and WLE groups. A clear difference in the metabolomic expression between the BLE, WLE, and YLE groups and the control group is displayed, where a significant cluster of metabolites was observed between the groups.

**Figure 3:**
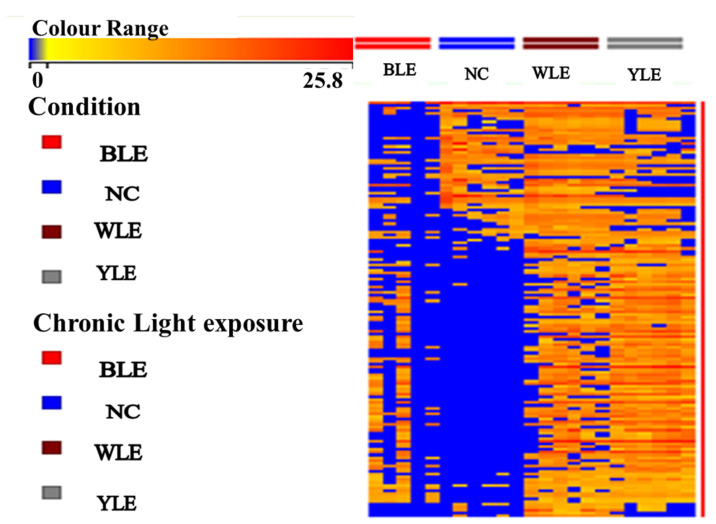
Hierarchical clustering analysis (HCA) by R version (version 3.6.3). HCA of the significant metabolites across the group were plotted in heat map. The color gradient illustrates the fold change (Fc3.1) in protein abundance between the four groups. The ratio of precursor to metabolite for each sample value is calculated, and significant metabolites are selected and plotted. Rows represent control (NC), blue light exposure (BLE), white light exposure (WLE), and yellow light exposure (YLE) groups, and metabolic column ratio. The color spectrum toward red represents the upregulation of metabolites and blue represents the downregulation, respectively. A clear separation of the metabolite’s alteration in the three exposure and control group conditions was observed, and the alteration becomes more obvious when significant metabolites have been considered.

Figure 3 represents the heat map of significant (243 molecules (*p* < 0.05)) metabolites. We observed a clearer upregulation of molecules in the BLE, WLE, and YLE groups than in the NC group. The BLE and WLE exposure groups have been vital in showing the specificity of these biomarkers to the vitreous. Some metabolites were perturbed in all groups. Untargeted metabolomics by LC-MS/MS revealed 20 biologically relevant metabolites in ocular fluids. Thus, we next sought to validate our findings using the targeted analysis to quantify the concentrations of these metabolites. As well as targeting the metabolites dysregulated in BLE, WLE, and YLE samples, we expanded the targeted analysis to include metabolites from related metabolic pathways to determine the biological relevance of our findings using the METLIN, Agilent pro, and HMDB databases. The focused analysis was carried out using authentic standards to obtain accurate fold changes in the metabolites in the extracted samples. It was confirmed that 20 metabolites were statistically significant.

To further understand the perturbed metabolomic alterations that develop over the light exposure period of 90 days in Wistar rats, we performed a one-way ANOVA, and these values were compared across the groups. The *p*-values < 0.05 are considered significant, and the significantly altered metabolites between the groups are presented in Table 1 using the Tukey HSD post-hoc test.

**Figure 4:**
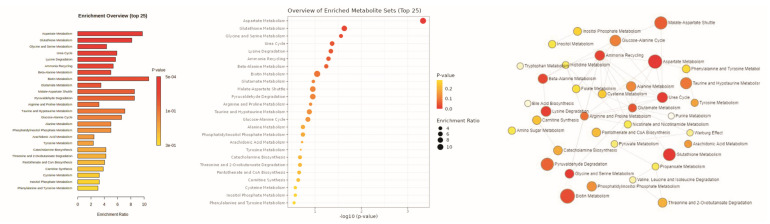
Metabolite set enrichment analysis: The aspartate, glycine, serine, and glutathione metabolic pathways are the most significantly affected in the upper panel; four upregulated FDR = 2.99 × 10^−1^ and 4/20; FDR = 1.00 in the lower panel; there are four dysregulated features out of 20; false discovery rate (FDR) = 1.00).

The pathway enrichment (Figure 4) analysis was performed for the highly significant top 20 metabolites in the NC, BLE, WLE, and YLE groups using the MetaboAnalyst 4.0 program. The analysis revealed that arginine, aspartate, glutamate, and taurine metabolites were the most perturbed pathways in light exposure conditions (Figure 4). The pathway analysis of the significant metabolites identified for the comparison among BLE revealed significant enrichment for aspartate (*p* = 0.0068), glutathione (*p* = 0.0263), glycine (*p* = 0.00436), glutamate (*p* = 0.054), leucine, and taurine (*p* = 0.007). Taurine and hypotaurine degradation (Figure 4) were the most perturbed pathways in BLE and WLE conditions. This augment growing evidence to suggest that photochemical metabolism is particularly compromised in LE animals, which potentially disrupts the taurine and hypotaurine dysregulation pathways.

## 4. Discussion

This study reveals that 90 days of (12:12 h) cyclic LED light exposure to a rat’s eye resulted in vitreous metabolomic alterations. These observations might be related to oxidative stress within the retinal tissue, consistent with the aforementioned findings of short exposure, especially for a few days to a few months [6,13,31,32].

The LC-MS study showed apparent changes in the vitreous metabolites after 90 days of light exposure (BLE, WLE, and YLE), and some of these changes, such as upregulated amino acids (AA) and lipids, were more intense in the BLE and WLE groups, while others, similar to the increase in levels of AA, were observed in the YLE group. Light-exposed vitreous showed several notable differences in the spreading silhouettes of the perceived metabolites between LED light exposure groups and normal controls of the vitreous fluid, predominantly amino acids and proteins. Inadvertently, the role of the altered metabolites and excitotoxic potential in light-induced damage in the vitreous is not well documented. It has been involved in a few ischemic-induced ocular disease conditions, including retinal vessel occlusion, glaucoma, and diabetic retinopathy [33,34]. The glutamate levels were altered in the BLE and WLE groups, which could also be a triggering factor for retinal apoptosis [35,36]. Glutamate is primarily mediated by overstimulation of the NMDA subtype of the glutamate receptor, triggering an increase in intracellular calcium and initiating a cascade of events that finally lead to cell death/apoptosis, depending on the glutamate levels [37]. The eccentricities in glutamate metabolism result in the elevation of the extracellular concentration of glutamate, which might increase the risk of excitotoxicity [35]. This indicates that a failure to uphold glutamate homeostasis might lead to ocular toxicity [35].

We noticed that metabolites of the vitreous fluid were dysregulated in the light exposure (BLE and WLE) groups, which could be due to a shift in glycolysis products, such as fructose 1,6 bisphosphate (F1,6BP, a product of glycolysis) and citrate, to the inner retina, while glucose 6 phosphate (G6P) was detected at high intensities both in the inner and outer retina [38]. The upregulated metabolites in the BLE and WLE groups could be due to metabolomic requirements to withstand or protect from light-induced damage. Studies have demonstrated the importance of the cytokines-induced protective mechanism of photoreceptors in light-induced damage against constant exposure [39,40] and stated that this could be due to the survival-promoting genes and proteins that might alter during retinal light adaptation to a new bright light environment in the BLE and WLE groups. The identified molecules from Alvarez et al., 2001, which can protect against light-induced damage, were upregulated contemporaneously [41].

Interestingly, de la Barca et al., 2017 [14], studied the effect of light stress on the retinal metabolomes and found that the mechanisms of light preconditioning (pre-exposure to moderate light before intense light) remained unknown, even though they seemed to have a self-protective effect. The light-induced changes in vitreous metabolites such as amino acids and lipids could likely involve nitric oxide-related signaling pathways [14,38]. In post-acute bright light exposure, the retinal proteins’ expressions were upregulated, in particular the glial fibrillary acidic protein, heme oxygenase, and heat-shock protein, as well as cytokines [40,42,43,44]. Under a constant light exposure condition, the endurance of the alteration of the vitreous metabolites and their concentration revealed that this could be an endogenous oscillator; it correlates with previous experiments on aqueous proteins [45]. The upregulated metabolite concentrations noticed in the light exposure groups (BLE and WLE) (Figure 3) could be related to light responses in ocular-bounded tissues or linked to cellular restoration [45] and antioxidant protection in retinal tissues [46]. Abundant proteins have remained to play a major role in modifying such light damage [47]. Our current experiment suggests that the degree of susceptibility to light damage in tissues adjoining the vitreous may also be contingent upon time and light dependence.

With the increase in the period of cumulative and constant light exposure for 90 days on vitreous fluid, the self-protective or reworking system in the light (12:12 light-dark cycle) period could be depleted and might lose the ability to compensate for the defending mechanism, and thus might fail to maintain the metabolomic structural integrity. Such mechanisms increase post-LED exposure if the compensatory mechanism fails due to light damage and might lead to lethal consequences to the biochemical composition of vitreous fluid.

Few studies have indicated that lipofuscin could be a possible mediator of the risk associated with long-term exposure to blue light-induced retinal damage [48,49]. When lipofuscin absorbs blue light, it produces ROS that eventually lead to oxidative stress and retinal damage [29]. This mechanism (lipofuscin) is directly related to the type of light and its spectral composition [50]. Thomas et al. reported that chronic blue light exposure could accelerate photoreceptor degeneration in an animal model based on a retinal degeneration study [51]. The lipofuscin levels in the retinal pigmented epithelium may alter with age, with low levels in young animals and high levels in old animals [52]. This could also be one of the confounding factors that accelerates retinal damage, along with chronic exposure to the blue and white components of the light spectrum. The presence of a self-protective mechanism diminishes with age [53]. Light exposure leads to oxidative stress, eventually resulting in the visual system no longer being able to protect the retinal architectures against chronic light, thus triggering irreversible vagaries in the visual system. Overall, the attained results can suggest the following sequences of events: the ROS produced by the high-energy light from a blue and white LED [54] and the altered vitreous metabolites by inducing protein and AA modification affecting the hypotaurine pathway. This observation suggests that photochemical metabolism is compromised in light-exposure rats, potentially disrupting taurine and hypotaurine metabolism that might impact neural signals in the retina (Figure 4). All three LED light sources used in this study had a large blue spectral contribution, which was evident from the overlapping results among the three groups, but the differences among the groups might have also been influenced by the red and yellow spectral contributions in the white and yellow LEDs. This study has some limitations. The results shown here were obtained in rats with cumulative and continuous LED light exposure. Their eyes are different from humans’ because they are nocturnal animals and do not have a macula, therefore, this cannot be directly applied to humans. Moreover, this study is qualitative, and inferences, such as the actual physiological effects, cannot be compared with humans.

## 5. Conclusions

Our findings state that chronic exposure to blue and white LED light alters the vitreous metabolites. The prolonged acute cumulative blue and white LED light exposure causes indemnities to the structural integrity of the vitreous. The self-protective or reworking system in the light-exposed visual system is dazed and deteriorates the ability to compensate for the self-protective mechanism.

## Figures and Tables

**Table 1:** Distinguish altered vitreous metabolites in NC, BLE, WLE, and YLE metabolite intensities of the most abundant and significantly altered amino acids, small molecules, and lipids detected in the vitreous of NC (*n* = 6) BLE (*n* = 6), WLE (*n* = 6) and YLE (*n* = 6). Statistically significant changes in metabolite intensity between the control and lens protection groups are represented by indicating (*p* < 0.001, *p* < 0.01, *p* < 0.05).

S.No	Mass	Metabolites	*p*-Value-ANOVA	Tukey HSD Post-Hoc Analysis					
				NC-BLE	BLE-WLE	BLE-YLE	NC-WLE	NC-YLE	WLE-YLE
1	89.0932	D-Alanine	**0.013**	**0.02**	1	0.753	**0.021**	0.146	0.773
2	105.0926	D-Serin	**0.009**	**0.091**	**0.016**	**0.014**	0.84	0.811	1
3	119.1192	L-Threonine	**<0.001**	**0.002**	0.503	0.629	**<0.001**	**<0.001**	0.997
4	125.147	Taurine	**0.006**	0.861	**0.006**	0.16	**0.032**	0.509	0.401
5	128.172	Lysine	**0.001**	**0.014**	0.733	0.89	**0.001**	**0.003**	0.989
6	132.1577	L-Asparagine	**0.011**	0.051	0.987	0.943	**0.025**	**0.016**	0.996
7	133.1027	D-Aspartic acid	**0.012**	0.122	0.975	**0.021**	0.25	0.818	**0.05**
8	147.13	L-Glutamic acid	**<0.001**	**<0.001**	0.509	0.857	**0.009**	**0.002**	0.926
9	153.1784	Dopamine	**0.009**	**0.091**	**0.016**	**0.014**	0.84	0.811	1
10	175.188	Citrulline	**<0.001**	**<0.001**	0.734	0.995	**0.002**	**<0.001**	0.596
11	219.237	Pantothenate	**0.017**	0.096	0.92	0.94	**0.026**	**0.03**	1
12	228.245	Hydroxyprolyl-Proline	**0.004**	0.053	0.619	0.975	**0.004**	**0.022**	0.851
13	231.249	L-Asparaginyl-L-Valine	**0.004**	0.979	0.994	**0.02**	0.999	**0.008**	**0.012**
14	231.296	α-(γ-Aminobutyryl)-Lysine	**0.02**	0.338	0.414	0.778	**0.017**	0.064	0.923
15	284.272	L-Glutamyl-L-Histidine	**0.038**	0.22	0.849	0.46	**0.049**	0.956	0.131
16	307.32	Glutathione	**0.012**	1	**0.019**	0.743	**0.02**	0.757	0.144
17	317.3398	L-Hydroxyprolyl-L-Tryptophan	**<0.001**	**0.002**	0.965	0.999	**0.001**	**0.001**	0.989
18	364.5619	2-Arachidonylglycerol	**0.041**	**0.067**	0.999	0.87	**0.05**	0.263	0.802
19	420.0956	Inositol 1,4,5-Trisphosphate	**0.029**	0.176	0.826	0.935	**0.033**	0.059	0.993
20	579.7895	Lysophosphatidylcholine	**0.011**	0.819	**0.01**	0.139	**0.066**	0.514	0.598

## Data Availability

The data presented in this study are available on request from the corresponding author. The data are not publicly available due to privacy.

## Supplementary Materials

The following supporting information can be downloaded at: https://www.mdpi.com/article/10.3390/metabo13010081/s1, Figure S1: Explains the light sources that were used for the experiment. The blue LED ((400–490 nm), BLE 1a), white LED ((380–780 nm), WLE 1b) and (yellow LED (400–780 nm) YLE 1c). The peak emission (420–470 nm) spectrum curves for the blue light were seen in white light similar to blue LED light and the peak was declined in the yellow light.
