# Supplementary material for: Effect of Light Emitting Diodes (LED) Exposure on Vitreous Metabolites-Rodent Study
Source: Metabolites. 2023 Jan 3;13(1):81. doi: 10.3390/metabo13010081 (PMC9861686; doi:10.3390/metabo13010081)
Supplement: Supplementary file 1 [file metabolites-13-00081-s001.zip › metabolites-2101115-supplementary.pdf]

Supplementary file

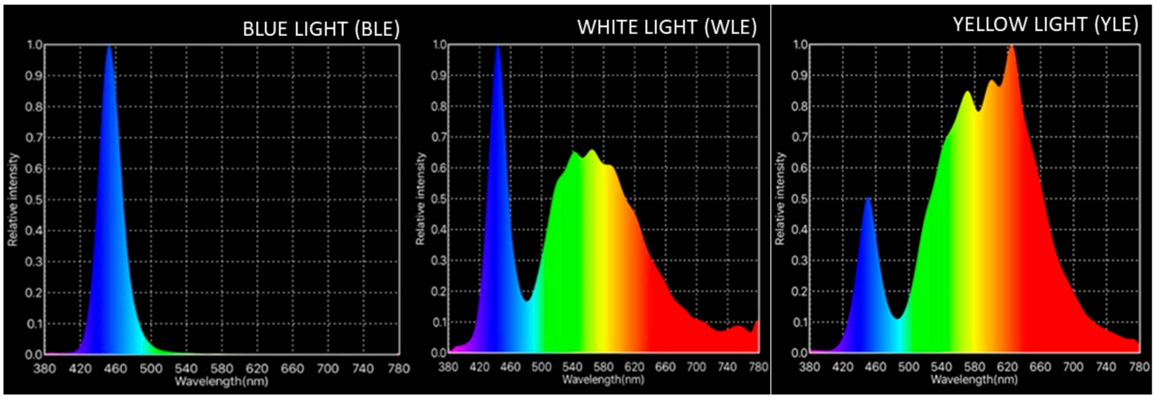

Figure S1. Explains the light sources that were used for the experiment. The blue LED. ((400–490 nm), BLE 1a:), white LED ((380–780 nm), WLE 1b:) and (yellow LED (400–780 nm) YLE 1c: The peak emission (420–470) spectrum curves for the blue light were seen in white light similar to blue LED light and the peak was declined in the yellow light.
